# Ultrafast microwave synthesis of rambutan-like CMK-3/carbon nanotubes nanocomposites for high-performance supercapacitor electrode materials

**DOI:** 10.1038/s41598-020-63204-3

**Published:** 2020-04-10

**Authors:** Ke Yan, Xin Sun, Shu Ying, Wen Cheng, Yu Deng, Zhong Ma, Yu Zhao, Xinran Wang, Lijia Pan, Yi Shi

**Affiliations:** Jiangsu Provincial Key Laboratory of Photonic and Electronic Materials, Collaborative Innovation Center of Advanced Microstructures, School of Electronic Science and Engineering, Nanjing University, Nanjing, 210093 China

**Keywords:** Materials for energy and catalysis, Nanoscale materials, Nanoscale materials, Energy storage

## Abstract

Ordered mesoporous carbon materials show great potential for electric double-layer supercapacitors because of their high specific surface area, designable pore structure, and tunable morphology. However, low graphitic crystallinity nature and poor contact between particles lead to their high inherent resistance, which limits the supercapacitance performance. Herein, we report on a hierarchically rambutan-morphological design of carbon composites with ordered mesoporous carbon as the core and carbon nanotubes as the shell, which significantly improve the electric contact between mesoporous carbon particles and promote the electrochemical performance. By an ultrafast microwave process in a household microwave heater under ambient condition, carbon nanotubes grow out from the pores of ordered mesoporous carbon and are dispersed on its surface like the whiskers of rambutan. As-synthesized ordered mesoporous carbon CMK-3/carbon nanotubes nanocomposites show significantly enhanced specific capacitance (315.6 F·g^−1^ at 1 A·g^−1^, as compared with 172.1 F·g^−1^ of CMK-3), high rate capability (214.6 F·g^−1^ at 50 A·g^−1^), and cycling durability (10,000 cycles, 99.32%). The structural design and microwave synthesis enable a facile preparation of the hybrid ordered mesoporous carbon CMK-3/carbon nanotubes nanocomposites, and show potential for easy and low-cost production of high performance electric double-layer supercapacitors materials.

## Introduction

In recent years, conflicts between energy-consuming and global ecology have urged researchers to develop new energy conversion and storage technologies with efficient, environmental-friendly, and sustainable features^1,2^. Supercapacitors (also known as Electrochemical capacitors) nowadays increasingly attract the attention of researchers and have been considered as promising devices for future energy storage applications, with the advantages of high power rating compared with batteries, large energy density compared with dielectric capacitors, and excellent charge-discharge stability^3–10^. Based on the energy storage mechanism, it can be divided into pseudocapacitors (typically produced with electro-active materials, such as transition metal oxides and conducting polymers), and electric double-layer supercapacitors (EDLCs, with non-redox materials, such as active carbon, porous carbon, etc.). For EDLCs, energy storage is achieved by electrostatic interaction of electron charge in the electrode and adsorbed ions in a Helmholtz double layer at the interface between electrode/electrolyte. Owing to the non-Faradaic process of charging/discharging, carbon-based EDLCs endow high reversibility and stability in aqueous electrolyte^11^. However, due to poor electric contact between active material particles and inefficient ion diffusion in the pore structures, relatively low energy density and poor rate performance hinder EDLCs practical applications and further development^12,13^.

To deal with these challenges, various materials were developed to improve the EDLCs performance, and carbon-based nanostructures were among the best of them^14–24^. Ordered mesoporous carbon CMK-3 was one of the promising materials because of the high surface-area-to-mass ratio mesopores (pore diameters between 2 and 50 nm), aligned channel for promoted ion diffusion, and modifiable surface morphology^25–28^. However, the low graphitic crystallinity nature of CMK-3 and ineffective electric contact between CMK-3 particles generally resulted in high inherent resistance of the electrodes which seriously restricted the charge and discharge efficiency, and thus limited the electrochemical performance of CMK-3 (usually exhibiting a specific capacitance of ~150 F·g^−1^)^29,30^. On the other hand, carbon nanotubes (CNTs) was considered as another potential EDLCs electrode material because of the unique electrical, mechanical, and chemical properties^31,32^. CNTs have superior electrical conductivity than amorphous carbon materials, which endow them with high electron transport efficiency and good rate performance; in addition, mechanical robustness of CNTs can reduce the cycle degradation of capacity during the charging and discharging process. Nevertheless, the CNTs electrodes generally have a specific capacitance of fewer than 100 F·g^−1^ because of the few effective pore structures for ion diffusion.

Designing and fabricating composites of CMK-3 and CNTs to effectively couple their advantages can be a good solution to the aforementioned challenges. There were some previous works reported on the physically mixing of CNTs and CMK-3 particles, in which CNTs acted as the conductive binder to enhance the electron transport. Some other works tried to use the conventional Chemical vapor deposition (CVD) process to synthesize CNTs on CMK-3 particles’ surface^33–36^. However, these approaches also suffered from the weakened binder connection and bad electric contact between CMK-3 and CNTs. Moreover, the CVD process was relatively hard to control, time-consuming, and high-cost. Nowadays, microwave techniques have been applied in synthesis and modification of nanomaterials for their fast, energy-saving, and controllable specialties^37–41^.

Herein, we proposed an ultrafast microwave synthesized rambutan-like CMK-3/carbon nanotubes nanocomposites with improved electric contact between mesoporous carbon particles and significantly enhanced supercapacitance performance. CMK-3 acted as the microwave-absorbing conductive layer, and ferrocene was filled in the nanochannels of CMK-3 to form nanoparticles. The local heating induced by microwave irradiation, within only 10~30 seconds, decomposed ferrocene into Fe nanoparticles and catalyzed the growth of CNTs out of the CMK-3 nanochannels. As-synthesized CMK-3/CNTs nanocomposites showed the morphology like rambutans with CNTs whiskers, which significantly enhanced the electric contact by 3D interconnecting routes between CMK-3 particles. Superior EDLCs performances were observed, such as enhanced specific capacitance (315.6 F·g^−1^ at a current density of 1 A·g^−1^), high rate capability (214.6 F·g^−1^ at 50 A·g^−1^) and excellent cycling stability (99.32% after 10,000 cycles). The results show that the ultrafast microwave synthesized CMK-3/CNTs nanocomposites have great potential for high-performance supercapacitor applications. The microwave synthesis and convenient precursors could provide with an ultrafast, easy procedure, and low-cost approach for massive commercial production of the supercapacitor electrode materials.

## Results and Discussion

### Formation mechanism of CMK-3/CNTs nanocomposites

**Figure 1:**
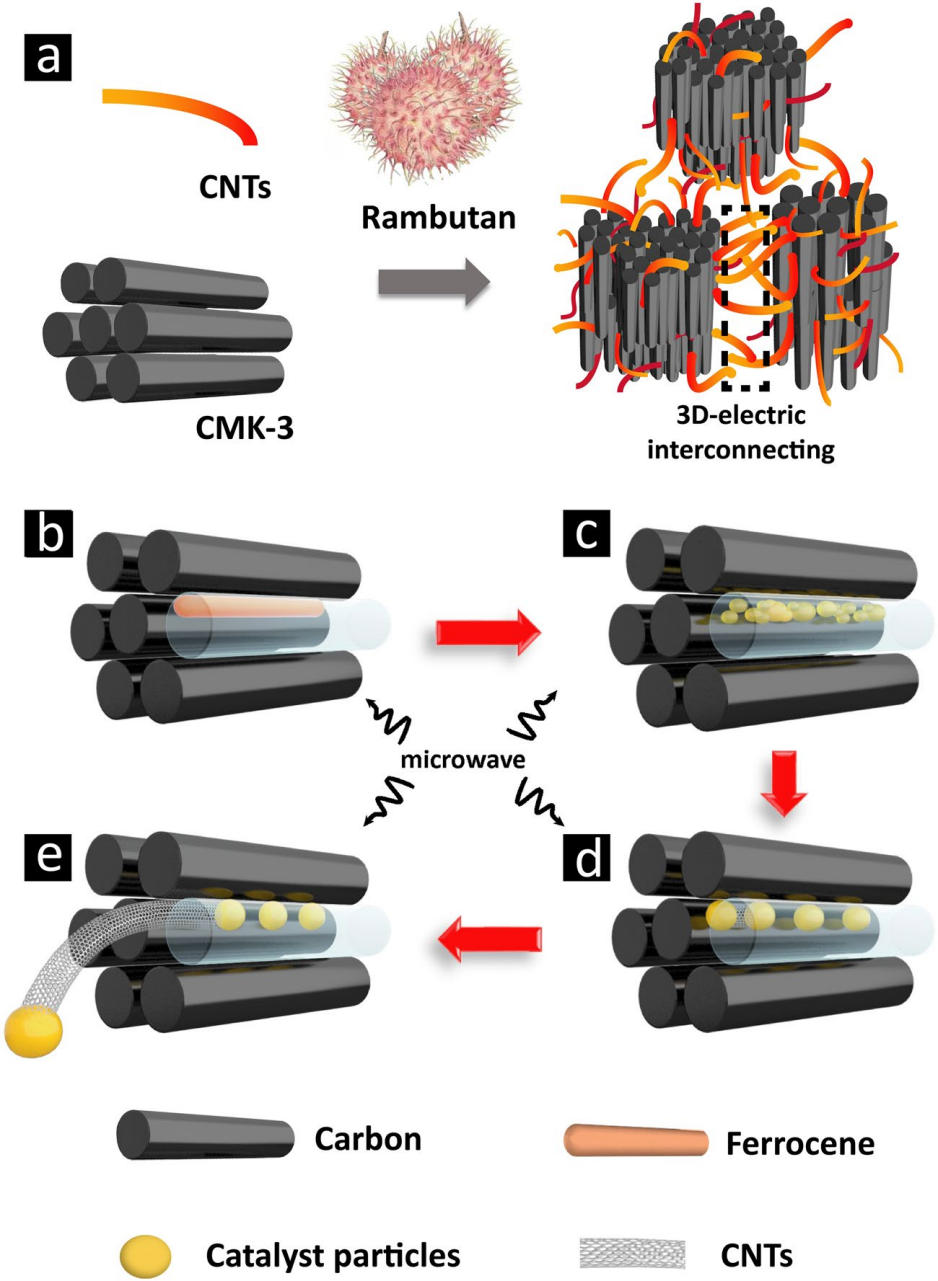
(**a**) Schematic illustration of CNTs, CMK-3 and rambutan-like CMK-3/CNTs nanocomposites. Growth mechanism of microwave-induced CNTs: (**b**) Ferrocene filled in the nanochannels of CMK-3; (**c**) Microwave-induced ferrocene decomposition; (**d**,**e**) Iron particles catalyzed growth of CNTs.

The structure of CMK-3/CNTs is schematically illuminated in Figure 1a. The synthesis process can be divided into two steps. In the first step, under microwave heating, ferrocene precursors trapped in the CMK-3 were decomposed into Fe catalysts with sizes precisely defined by the nanochannels of CMK-3 (Figure 1b–d). The mixture surface temperature got up to 390 °C in seconds as measured by thermal imager (Fig. S1) and the instantaneous local temperature was estimated over 1000 °C due to the heat release of sparks and arcs according to previously reported^42^. The second step (Figure 1d,e) is the tip-growth of CNTs catalyzed by nano-sized Fe particles, which serve as both nucleation centers and catalysts of cyclopentadienyl (from the decomposition of ferrocene) (Fig. S2)^43,44^.

The CMK-3 powder acted as good heating and microwave absorption material, while it was found that the temperature of ferrocene would not raise under microwave irradiation due to its nonpolar nature^45^. Microwave radiation made the electric dipoles and charges of conductive material oscillate in translational motion with high frequency, and the kinetic energy produced by molecular friction turned into heat intensively and rapidly^46,47^. Hence, the high electrical conductivity of the CMK-3 helped the precursor absorb microwave irradiation and thus a large amount of heat was generated in a short time period. In addition, due to the low microwave penetrating depth (i.e. the ability of a material to be heated by microwave irradiation) of graphite^42,48^, the microwave heating was produced on the CMK-3 surface within several microns. We observed no heating, sparks or arcs generation of other mesoporous materials such as SiO_2_ molecular sieves under microwave radiation for the much higher penetration depth of silicon materials than graphite.

### Structure and morphology of CMK-3/CNTs nanocomposites

**Figure 2:**
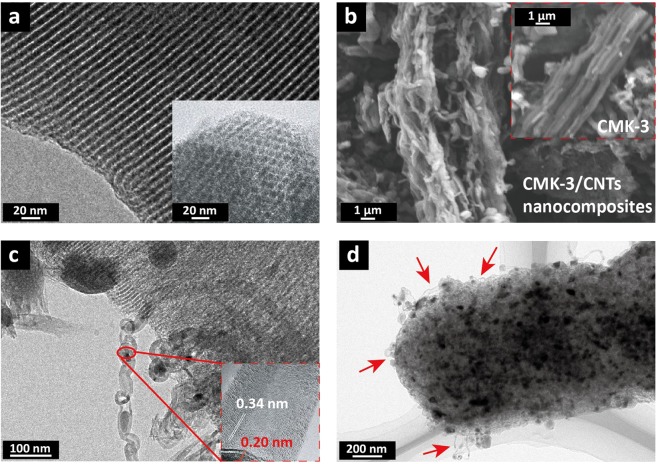
(**a**) TEM image of pristine CMK-3 nanochannels; Inset, hexagonal ordered mesostructured view of CMK-3; (**b**) SEM image of as-synthesized CMK-3/CNTs nanocomposites, of which two particles are connected by CNTs wrapped around CMK-3; Inset, pristine CMK-3 particles; (**c**) TEM images of CNTs in bundles synthesized by the microwave process; Inset, electron diffraction pattern of CNTs and catalyst particles; (**d**) TEM images of CNTs protruding from the mesoporous nanochannels of CMK-3.

Microwave-induced CNTs was generated on the surface of CMK-3 due to the aforementioned local-heating effects. Figure 2a shows the Transmission electron microscopy (TEM) image of a single CMK-3 particle with parallel and periodic pore structures. It has pore diameters of 3.8 nm–4.0 nm, and carbon cylinders’ diameter of ~ 7.3 nm. Figure 2b shows the Scanning electron microscopy (SEM) image of as-synthesized CMK-3/CNTs nanocomposites, of which two particles are connected by CNTs wrapped around CMK-3. As a comparison, the pristine CMK-3 particles show bare ordered rod-like morphology without additional structure on the surface. TEM image in Figure 2c shows that well-crystallized CNTs pile up, forming bundles from the pore and at the end of the CMK-3. The inset of Figure 2c presents good graphitization of CNTs with ~0.34 nm layer distance and Fe nanoparticles (the (1 1 0) plane of the Fe (JCPDS06-0696)) encapsulated in the nanotubes as catalyst (from the decomposition of ferrocene) which correlate well with the previous reports^49,50^.

Figure 2d shows highly dense of well-entangled CNTs (marked by red arrows) distributing on the CMK-3 particles and the bundle diameter ranges from ~ 1 nm to ~ 10 nm (Fig. S3). Due to the efficient *in suit* growth of CNTs, the adhesion and interactions between CNTs and substrates were improved to couple eligible CMK-3/CNTs nanocomposites^51^.

**Figure 3:**
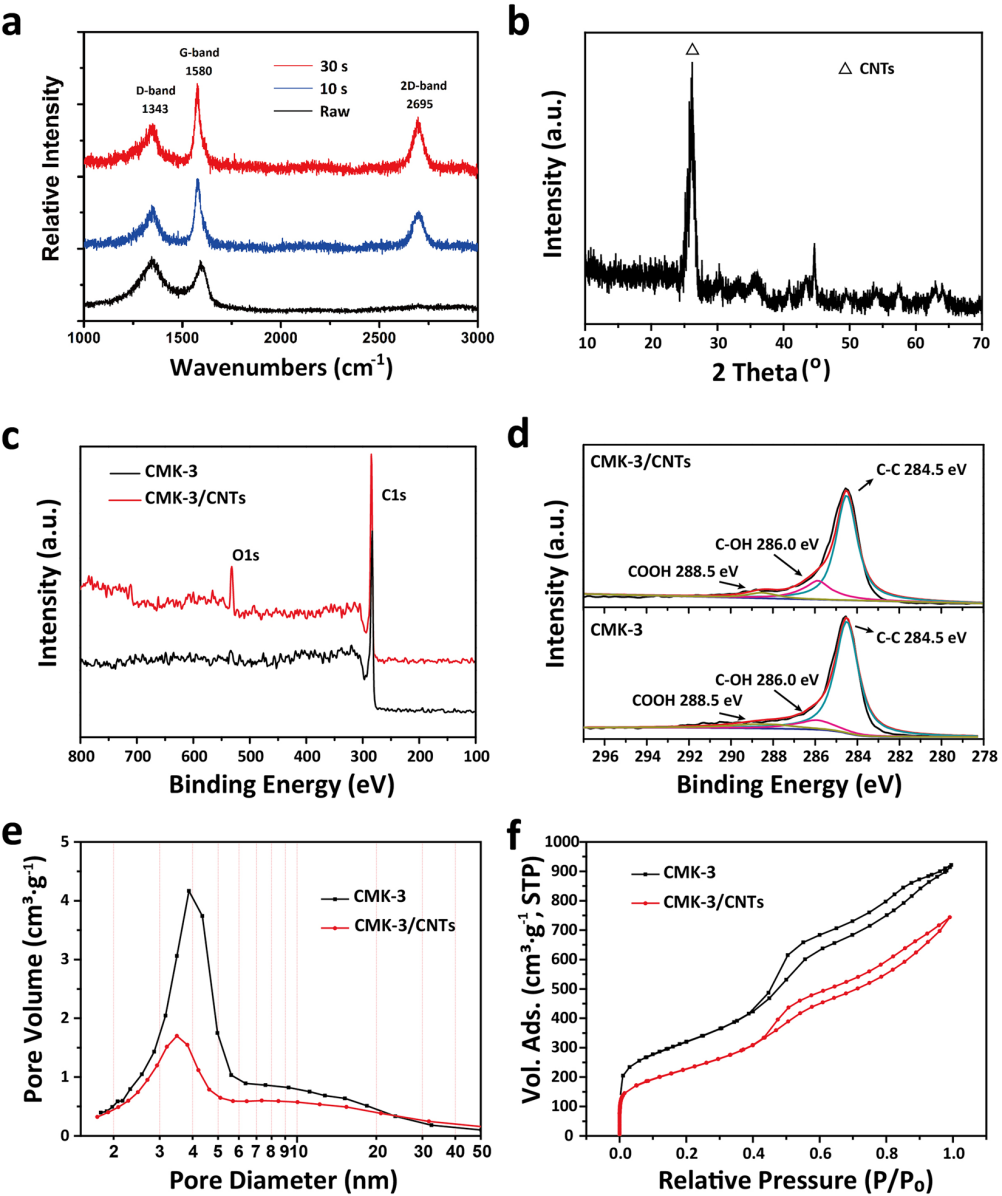
(**a**) The Raman spectra of the composites with different microwave time; (**b**) The XRD patterns of the CMK-3/CNTs nanocomposites; (**c**) Full-range XPS spectra of CMK-3 and CMK-3/CNTs nanocomposites; (**d**) C 1 s XPS spectra of CMK-3/CNTs nanocomposites and CMK-3 before microwave process. (**e**) The pore size distributions of CMK-3/CNTs nanocomposites and pristine CMK-3; (**f**) The N2 adsorption/desorption isotherm curves of CMK-3/CNTs nanocomposites and pristine CMK-3.

The Raman spectra confirm the formation and coupling of CNTs. The measurements were performed at a laser excitation line of 514 nm and the spectra were shown in Figure 3a. As-synthesized CMK-3/CNTs nanocomposites exhibit D-band (with peaks of 1100–1400 cm^−1^) which is caused by graphite layer disorder or defects; a G-band (with peaks of 1580–1600 cm^−1^) which is related to sp^2^ bond of carbon atoms, denoting the existence of CNTs and good graphitic quality; and a 2D-band (with peaks double the wavelength of D-band) which comes from a second-order double resonant process. The intensity ratio of D-band to G-band (I_D_/I_G_) refers to the amount, crystallized degree and purity of CNTs and the decrease of I_D_/I_G_ value proves the increase of CNTs in our sample^52^. As shown in Figure 3a, after microwave treatment for 10 seconds, as-synthesized nanocomposites showed stronger G-band and weaker D-band compared with pristine CMK-3. The I_D_/I_G_ value of pristine CMK-3 and 10-seconds CMK-3/CNTs nanocomposites were 1.08 and 0.60, respectively. With the microwave treating time increasing from 10 s to 30 s, G–band peaks were further promoted while the I_D_/I_G_ value was further reduced to 0.50. However, when treated for more than 30 s microwave radiation, no obvious enhancement of I_D_/I_G_ value was observed because synthesis finished within just 30 s and more reaction time brought no extra CNTs formation. Besides, the I_D_/I_G_ values of CMK-3/CNTs nanocomposites synthesized with different concentration of ferrocene are shown in Table S1 with Atomic absorption spectrometry (AAS), showing that the most CNTs-productive approach of the synthesis process is the use of 80 wt.% ferrocene-saturated benzene solution.

The X-ray diffraction (XRD) patterns of the CMK-3/CNTs nanocomposites are shown in Figure 3b. The XRD patterns show the crystallographic structure of the sample. The diffraction peak of 26.4°is assigned to the (002) reflection of graphite. The XRD patterns of the CMK-3/CNTs nanocomposites before acid treatment are shown in Fig. S4A. The Small-angle X-ray scattering (SAXS) patterns of CMK-3 and CMK-3/CNTs nanocomposites are shown in Fig. S4B which revealed their well-ordered mesoporous structures with unit cell parameters calculated to be ~11.3 nm. Energy dispersive X-Ray spectroscopy (EDX) and X-ray photoelectron spectroscopy (XPS) were used to further characterize as-synthesized CMK-3/CNTs nanocomposites. EDX pattern (Fig. S5) is sensitive to trace amounts of elemental content in the CMK-3/CNTs nanocomposites and thus identify the purity. The elemental contents of CMK-3/CNTs nanocomposites are C, O, and Fe (Cu peaks caused by TEM copper net) corresponding to previous measurements that no other elements are introduced within the synthesis process. XPS is conducted to analyze the chemical bond types and the functional groups on the CMK-3/CNTs nanocomposites. The full-range XPS spectra of CMK-3 and CMK-3/CNTs nanocomposites are presented in Figure 3c. It shows that C 1 s and O 1 s peak are enhanced by the formation of CNTs^53^. As shown in Figure 3d, the C 1 s peaks are resolved into three peaks (at 284.6 eV, 286 eV, and 288.5 eV, representing C-C/C = C, C‒OH and COOH, respectively)^54^. CMK-3/CNTs nanocomposites (top) exhibit enhanced C‒OH and COOH peaks, compared with pristine CMK-3 (bottom) which is ascribed to the oxidization and heating environment within the microwave synthesis process. Also, hydrophilic property of the CNTs surface could be promoted through the microwave oxidization and heating treatments for the functional groups’ introduction. In Figure 3e, the distributions of pore sizes are given, derived from desorption data using the Barrett−Joyner−Halenda (BJH) model. It shows most of the pore diameters fall into 2.0-10.0 nm and the diameter peaks of CMK-3 and CMK-3/CNTs nanocomposites are calculated to be around 3.9 nm and 3.6 nm. The average pore diameters are narrowed after the presence of CNTs. The N2 adsorption-desorption isotherm curves in Figure 3f indicate a sharp uprising below *P/P*_0_ = 0.02 and a hysteresis loop above *P/P*_0_ = 0.6, corresponding to type III curve and type H-3 loop in IUPAC classification which are characteristics of uniform mesoporous particles^55^. A large specific surface area (1108.74 cm^2^**·**g^−1^) and pore volume (1.58 cm^3^·g^−1^) were obtained from the CMK-3 which provided active sites and channels for the adsorption and diffusion of electrolyte ions. For the CMK-3/CNTs nanocomposites, the specific surface area and pore volume were changed to 801 m^2^·g^−1^ and 1.16 cm^2^·g^−1^, respectively. The decrease of both specific surface area and pore volume and the changes of pore size distribution are probably owing to the microwave synthesized CNTs located between the pore of CMK-3. However, this slight decrease of the specific surface area and pore volume have little effect on the capacitive properties of the CMK-3/CNTs nanocomposites as we can see in electrochemical measurements. The results further confirm that the capacitance has more than a simple linear relationship with the surface area^56^. So this approach to promote the contact and electrical conductivity between CMK-3 particles is effective towards enhanced electrochemical properties, instead of simply modifying the pore structures.

Differential Scanning Calorimetry (DSC) measurement was carried out with a temperature range of 20 °C to 1000 °C. The DSC curve of precursor shows 1 main peak at about 440 °C and 2 shoulder peaks at 360 °C and 530 °C (Fig. S6A). It is supposed that the main peak is the decomposition of amorphous carbon and the shoulder peak at 360 °C denotes the ferrocene decomposition temperature^57^. Thermogravimetric analysis (Fig. S6B) showed that as the temperature was lower than 400 °C, the mass of the mixture kept losing for the decomposition of amorphous carbon. The weight loss (~ 50%) observed in the temperature ranging from 400 to 600 °C is mainly due to the decomposition of CNTs. When the temperature exceeds 600 °C, the weight remains for the little residue of the iron compounds.

### Electrochemical performances of CMK-3/CNTs nanocomposites

The electrochemical performances of the microwave-induced CMK-3/CNTs nanocomposites were investigated by cyclic voltammetry (CV), galvanostatic charge/discharge (GCD), and electrochemical impedance spectroscopy (EIS) tests. All the three-electrode tests were performed in 1 M H_2_SO_4_ aqueous solution as the electrolyte. The mass loading of the active materials on the individual electrode was about 5 mg.

**Figure 4:**
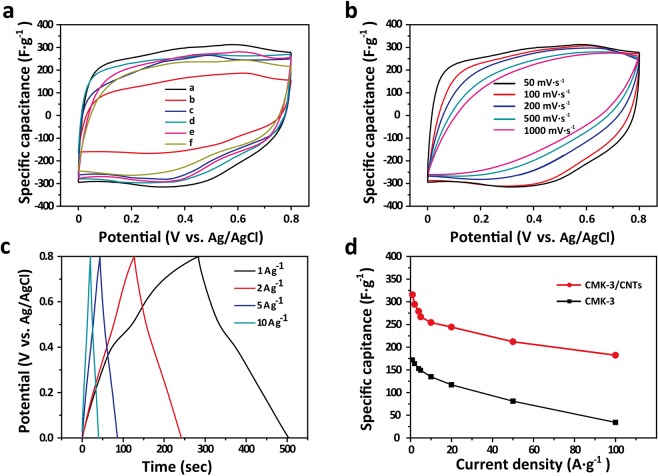
(**a**) CV curves of CMK-3/CNTs-x% at scan rate of 50 mV·s-1: **a**. CMK-3/CNTs-80%, **b**. pristine CMK-3, **c**. CMK-3/CNTs-100%, **d**. CMK-3/CNTs-90%, **e**. CMK-3/CNTs-70%, **f**. CMK-3/CNTs-60%; (**b**) CV curves of CMK-3/CNTs-80% at various scan rate; (**c**) GCD measurement of CMK-3/CNTs-80% at current density ranging from 1 A·g-1 to 10 A·g-1; (**d**) Specific gravimetric capacitance of CMK-3/CNTs-80% and pristine CMK-3 at different charge/discharge current densities.

In the synthesis step, 60 wt.%, 70 wt.%, 80 wt.%, 90 wt.% and 100 wt.% ferrocene-saturated benzene solution were marked with CMK-3/CNTs-60%, CMK-3/CNTs-70%, CMK-3/CNTs-80%, CMK-3/CNTs-90% and CMK-3/CNTs-100%. Figure 4a presents the CV curves (capacitance from current divided by the scan rate) of the CMK-3/CNTs-X% composites at a scan rate of 50 mV·s^−1^. It demonstrates that the CV curves are rectangular shapes which are the symbol of EDLCs. It is found from the CV curves that, compared with pristine CMK-3, the CMK-3/CNTs nanocomposites exhibit much enhanced specific capacitance. Among different precursor ferrocene concentrations, CMK-3/CNTs-80% exhibits the best electrochemical behavior. The high density of ferrocene would produce an abundant amount of catalyst thus enhancing the CNTs synthesis efficiency but the excessive content of ferrocene may cause the large bulk of Fe particles which hinder the CNTs catalysis process and even cause aggregation to block the nanochannels. After that, we investigated the CV curves of CMK-3/CNTs-80% at various scan rates ranging from 50 mV·s^−1^ to 1000 mV·s^−1^ as shown in Figure 4b. The curves remain its shape even at highest scan rates, indicating rapid ion diffusion and electron transport efficiency. The mesopores in CMK-3 were responsible for adsorbing ions from the electrolyte and shortening the ion diffusion pathways. CNTs were utilized to effectively build electrons interconnecting pathways in the nanocomposites thus reducing the internal resistance and enhancing the rate capability. In Figure 4c, GCD measurements at a current density ranging from 1 A·g^−1^ to 100 A·g^−1^ are given. The galvanostatic charge and discharge curves are nearly symmetric and the charging time is close to the corresponding discharging time. We further calculated the specific capacitance at various current densities shown in Figure 4d to analyze the rate capability of CMK-3/CNTs nanocomposites. The specific capacitance maintains 81% at a current density of 10 A·g^−1^ and even 69% at 50 A·g^−1^, indicating the prominent rate performance of CMK-3/CNTs nanocomposites. A comparison of the specific capacitances and rate capabilities of this work with recent work in regard to EDLCs or mesoporous carbon supercapacitors are shown in Table 1. It illustrates the comparable capacitive performance of CMK-3/CNTs nanocomposites in this work.

**Table 1:** The specific capacitances and rate capabilities of this work compared with other work about EDLCs or mesoporous carbon supercapacitors.

Materials	Specific capacitance	Rate capability
This work	315.6 F·g^−1^ at 1 A·g^−1^	214.6 F·g^−1^ at 50 A·g^−1^ (69% retention)
OMC/CNTs nanocomposite^58^	338.1 F·g^−1^ at 1 A·g^−1^	38% retention at 50 A·g^−1^
CNTs and activated high surface area carbon^59^	200 F·g^−1^ at 1 A·g^−1^	NA
Graphene Hydrogels^60^	220 F·g^−1^ at 1 A·g^−1^	64% retention at 100 A·g^−1^
Mesoporous carbon spheres/RGO sheets^61^	41.5 F·g^−1^ at 1 A·g^−1^	85% retention at 25 A·g^−1^
Flower-like and hierarchical porous carbon materials^62^	294 F·g^−1^ at a scan rate of 2 mV·s^−1^	71% retention at 500 mV·s^−1^
Hollow carbon spheres anchored on carbon nanotubes^63^	201.5 F·g^−1^ at 0.5 A·g^−1^	69% retention at 20 A·g^−1^

**Figure 5:**
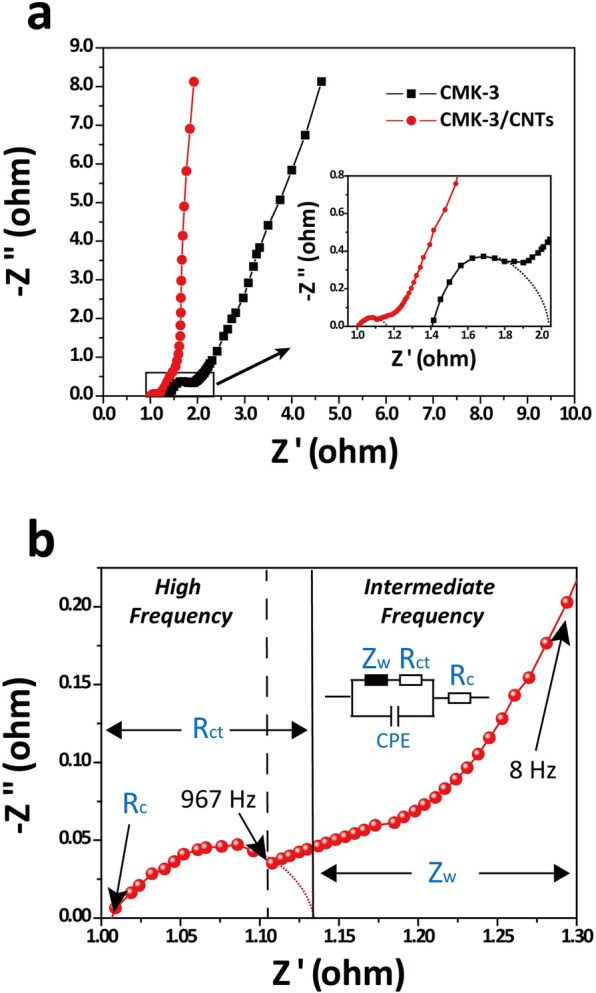
(**a**) Nyquist plots of the CMK-3/CNTs nanocomposites and pristine CMK-3; Inset, the expanded image of their high-frequency region; (**b**) Nyquist plots of the CMK-3/CNTs nanocomposites and equivalent circuit model of the EDLCs electrodes.

The EIS measurements of CMK-3/CNTs nanocomposites were performed at a frequency ranging from 0.01 Hz to 10 kHz. It is considered as a powerful complementary technique to investigate interfacial resistance between the electrode and the current collector, the ion adsorption and diffusion resistance and electron transport resistance of the electrode material. Nyquist plots of the CMK-3/CNTs nanocomposites and pristine CMK-3 are shown in Figure 5a. In the low-frequency part, CMK-3/CNTs nanocomposites curve exhibit more straight and vertical line compared with that of pristine CMK-3, indicating higher ion diffusion efficiency as ideal EDLCs. Figure 5b shows the equivalent circuit model of the EDLCs cell in high-frequency and intermediate-frequency part. The CPE represents EDLCs constant phase element and Z_w_ represents Warburg impedance. The intersection point value of Z’ axis (R_c_) is determined by the electrolyte conductivity, the electronic resistance of the electrode and the contact resistance at the interface between active materials and current collectors. R_c_ of CMK-3/CNTs nanocomposites is ~1.01 Ω lower than that of pristine CMK-3 (~1.4 Ω) because the conductive CNTs pinned to and wrapping on the CMK-3 particles facilitate the interfacial electric contact between the electrode material and the current collector^58^. Moreover, the CNTs bridge 3D network with each other and build up additional conducting routes between CMK-3 particles thus reducing the charge transfer resistance (R_ct_). Semi-circle diameter at high-frequency part is a symbol of charge transfer resistance (R_ct_) and the specimens of CMK-3/CNTs nanocomposites have lower R_ct_ (~0.12 Ω) compared with pristine CMK-3 (~0.62 Ω) indicating evidently enhanced electrical connection between well-ordered mesoporous structures. As a result, more efficient ions adsorption/diffusion and faster charge transport between the CMK-3 particles give rise to the improved electrochemical performances of CMK-3/CNTs nanocomposites. Comparison of as-synthesized CMK-3/CNTs nanocomposites with prstine CMK-3 as electrode materials are shown in Table 2.

**Table 2:** Comparison of as-synthesized CMK-3/CNTs nanocomposites with CMK-3.

Sample	*S*_BET_^a^ (cm^2^ ∙ g^-1^)	*a *^b^ (nm)	*R*_*c *_^c^ (ohm)	*R*_*ct*_^d^ (ohm)	*C*_*m *_^e^ (F ∙ g^-1^)	*E *^f^ (Wh∙kg^−1^)
CMK-3	1109	11.3	1.40	0.62	172.1	12.1
CMK-3/CNTs	801	11.3	1.01	0.12	315.6	21.7

^a^Surface area calculated by the (Brunauer–Emmett–Teller) BET method

^b^Cell parameters

^c^Intersection point value of Z’ axis

^d^Charge transfer resistance

^e^Specific capacitance at a current density of 1 A ∙ g^−1^

^f^Energy density at a current density of 0.5 A ∙ g^−1^

The cycle life of CMK-3/CNTs nanocomposites was tested at a current density of 10 A·g^−1^ to investigate its capacitance stability through the repeatedly charging-discharging process. After 5000 cycles, the capacitance retention was 96.8% as shown in Fig. S7. The slight increase of capacitance to 102.5% could be contributed by the activation process by acid electrolyte gradually infiltrated into all accessible mesoporous structures^64^. The degradation of the functional groups and dissolved oxygen in the electrolyte might lead to the slight loss of capacitance. Despite that, the nanocomposites showed remarkable cycling stability which corresponds to the inherent advantage of carbon-based electric double-layer supercapacitors.

### Electrochemical performances of CMK-3/CNTs symmetric EDLCs

**Figure 6:**
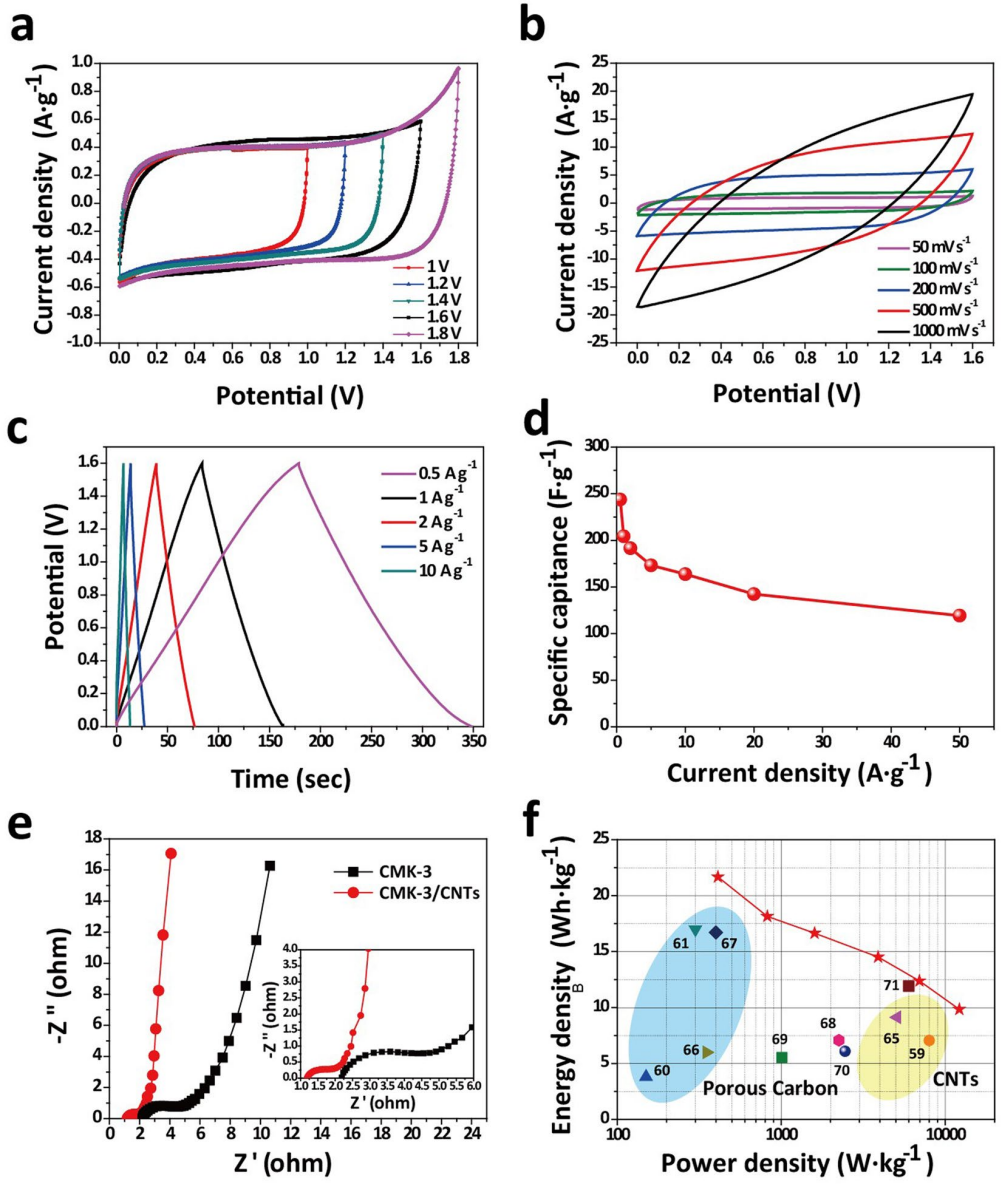
Electrochemical performance of CMK-3/CNTs nanocomposites symmetric EDLCs cell in a two-electrode system (1 M Na2SO4 as aqueous electrolytes at room temperature): (**a**) CV curves of the symmetric EDLCs cell at different operation voltage windows (scan rate 20 mV·s-1); (**b**) CV curves of the symmetric EDLCs cell at different scan rates; (**c**) GCD measurement of the symmetric EDLCs cell at different charge/discharge current densities; (**d**) The specific capacitance versus different charge/discharge current densities; (**e**) Nyquist plots of the CMK-3/CNTs and pristine CMK-3 based symmetric EDLCs cell; Inset, magnification of the high-frequency region; (**f**) Ragone plots of the symmetric EDLCs cell and comparison with recently reported high-performance symmetric supercapacitors in aqueous electrolyte.

To further investigate the superior properties of the CMK-3/CNTs nanocomposites, symmetric electrodes EDLCs cell were assembled and 1 M Na_2_SO_4_ aqueous solution was applied as the neutral electrolyte. The higher operating voltage window could be reached in Na_2_SO_4_ aqueous electrolyte due to over-potential for hydrogen evolution. As is shown in Figure 6a, expanded voltage range of 0–1.6 V was conducted, the CV curves kept rectangular shape at a scan rate of 20 mV·s^−1^, suggesting ideal EDLCs behavior. But 0–1.8 V voltage would result in sharp current density increase due to the polarization of electrode and decomposition of electrolyte in the charge/discharge process. CV curves of the symmetric EDLCs cell were attained at scan rates from 50–1000 mV·s^−1^ in the voltage range of 0–1.8 V (Figure 6b). As is shown in Figure 6c, GCD measurement at a current density ranging from 0.5 A·g^−1^ to 10 A·g^−1^ indicated symmetric charge/discharge time and no obvious polarization or IR drop at 1.8 V. The specific capacitance of single electrode (*C*_*sp*_) in symmetric EDLCs cell were calculated and shown in Figure 6d. *C*_*sp*_ of 243.81 F·g^−1^ at 0.5 A·g^−1^ and 119.18 F·g^−1^ at 50 A·g^−1^ suggest good rate capability of the symmetric cell due to rapid ion diffusion and charge transport of the CMK-3/CNTs electrode. In Figure 6e, Nyquist plots at the low and high-frequency region of the symmetric EDLCs cell were obtained. Compared with pristine CMK-3 electrodes symmetric cell, CMK-3/CNTs nanocomposites electrodes symmetric cell showed decreased internal resistance and enhanced charge transport efficiency. Ragone plots of the cell shown in Figure 6f demonstrate superior specific energy and power densities values of CMK-3/CNTs nanocomposites symmetric EDLCs cell, compared with previously reported literature of carbon-based composites, porous carbon and CNTs as electrodes^59–61,65–71^.

**Figure 7:**
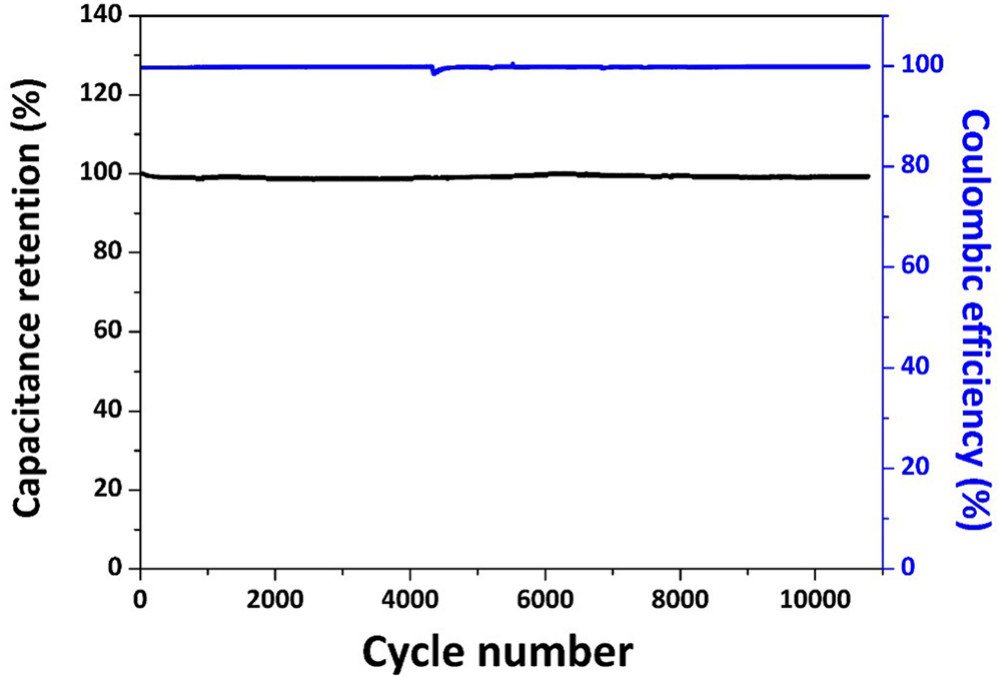
Cycling performance of symmetric EDLCs cell based on CMK-3/CNTs nanocomposites in a two-electrode system at current density of 10 A·g-1.

The capacitance retention and the Coulombic efficiency of the symmetric EDLCs cell were measured at charge/discharge current density of 10 A·g^−1^ for more than 10,000 cycles. As is shown in Figure 7, long-term stability was demonstrated that the Coulombic efficiency keeps around 100% and the capacitance remains ~ 99.32% after 10, 000 cycles. In addition, the pore and channel size of CMK-3 is also editable by controlling the reaction temperature during the synthesis process of SBA-15 or by adding boric acid during the CMK-3 synthesis process (see Supplementary Information), to meet extensive application requirements of active materials for energy storage and catalysis. We made a prototype symmetric supercapacitor with as-synthesized CMK-3/CNTs composites as electrodes to power a red light-emitting diodes (LED) light. The schematic illustration and photograph of the supercapacitor prototype were shown in Fig. S8.

## Conclusions

In conclusion, rambutan-like CMK-3/CNTs nanocomposites were synthesized by an ultrafast microwave heating approach for the first time. A 10–30 seconds’ microwave heating was conducted to catalyze i*n-suit* CNTs growth within the nanochannel of CMK-3. As-synthesized CNTs interconnecting between mesoporous CMK-3 particles can efficiently bridge 3D conducting networks. This characteristic structure contributed to the promotion of electron transport efficiency, pore connectivity and effective surface area for ion adsorption and diffusion. Therefore, the CMK-3/CNTs electrodes demonstrated superior electric double-layer capacitive performance, such as enhanced specific capacitance (315.6 F·g^−1^ at 1 A·g^−1^, as compared with 172.1 F·g^−1^ of pristine CMK-3), high rate capability (214.6 F·g^−1^ at 50 A·g^−1^) and excellent cycling stability (99.32% after 10,000 cycles). In the 2-electrode system, as-made symmetric electrodes cell demonstrated exceptionally high energy density and high power density along with long and stable cycle life. The microwave synthesis approach of CMK-3/CNTs nanocomposites shows great potential for ultrafast, easy and relatively low-cost production of the high-performance carbon-based electrode material for supercapacitors. We also envision our microwave approach as a time- and energy-saving method for varieties of high property nanocomposite materials’ synthesis and production which would have promising applications in energy storage, sensors, catalysis, etc.

## Methods

### Microwave synthesis of CMK-3/carbon nanotubes nanocomposites

In a typical process, 0.01 g CMK-3 powder (purchased from XF Nano, China) (typical synthesis process shown in Supplementary Information) were added into 5 mL 80 wt.% saturated solution of ferrocene (99.5%, Alfa Aesar) in benzene (99.5%, Shanghai Lingfeng) followed by 6 hrs of stirring using a magnetic stirrer and then stand still for another 6 hrs to let ferrocene fill in the nanochannels of CMK-3 by capillary force. After removal of redundant upper liquid, the mixture was placed in a quartz boat for air dry at room temperature for 24 hrs. Subsequently, a 30-seconds microwave process with the power of 700 W was applied to heat the precursor by a household microwave heater (Galanz P70D20TP-C6, China) and under ambient condition. It was observed that continuous arcs and sparks appeared on the surface of the precursor with the temperature rising rapidly. After obtaining relatively fluffy black powder, purification was conducted by washing it with benzene, acid treatment with HCl solution (∼37 wt.%) and then washing it by deionized water until the pH reached 7. Finally, the CMK-3/CNTs nanocomposites were gained after dried in vacuum at 60 °C overnight. In electrochemical measurement, 60 wt.%, 70 wt.%, 80 wt.%, 90 wt.% and 100 wt.% ferrocene-saturated benzene solution were used in the initial synthesis step, and the other conditions remained unchanged.

### Characterization and measurement

Thermal imaging was performed with Fotric-222s thermal imager. Scanning electron microscopy (SEM) was carried out by a JSM-7000F and transmission electron microscopy (TEM) was performed using a JEM-200CX. Thermogravimetric analysis (TGA) and differential scanning calorimetry (DSC) measurement were carried out in a temperature range of 20–1000 °C in air with a heating rate of 10 °C·min^−1^ using a Pyris 1 DSC thermal analyzer. Powder X-ray diffraction (XRD) analysis was conducted in an X’TRA with Cu Kα radiation from 3° to 70° at a step size of 0.02° to inspect the crystalline morphology. Small-angle X-ray scattering (SAXS) was conducted a by SAXSLAB Ganesha model with sample-to-detector distance from 100 mm to 1500 mm. Raman spectra were analyzed using a DU420A-0E-325, HORIBA Jobin Yvan. X-ray photoelectron spectroscopy (XPS) was measured on the PHI 5000 Versa Probe. Nitrogen adsorption-desorption isotherms were collected at 77 K using an ASAP 2020. The Brunauer–Emmett–Teller (BET) method was utilized to calculate the specific area and the pore volume. The pore size distribution was analyzed by the Barrett−Joyner−Halenda (BJH) method. Atomic Absorption Spectroscopy (AAS) was measured by a Hitachi 180–80.

### Electrochemical measurement

The electrochemical measurements were conducted on a CHI660C electrochemical workstation (Shanghai Chenhua Instrument Corporation, China). In a three-electrode system, the individual electrode was tested using 1 M H_2_SO_4_ aqueous solution as the electrolyte. The potentials were measured using Ag/AgCl (in sat. KCl) as the reference electrode and Pt wire as a counter electrode, respectively. The working electrodes were made with 1 cm×1 cm carbon cloths as current collectors. A certain amount of as-prepared CMK-3/CNTs nanocomposites was added into isopropanol and then ultrasonicated for 15 min to promote homogeneity. The slurry was then coated onto the carbon cloth current collector and then dried at 80 °C for 6 hrs to get the working electrode. Cyclic voltammetry (CV) and galvanostatic charge/discharge (GCD) measurements were conducted at various scan rates in the potential ranging from 0 to 0.8 V. The electrochemical impedance spectroscopy (EIS) were measured in frequency loop ranged from 0.01 Hz to 10 kHz with a voltage amplitude of 5 mV. The cycling stability was tested at a current density of 10 A·g^−1^ for 5000 charging/discharging cycles. ${C}_{m}$ (denotes the specific capacitances) were calculated according to the equation:

$${C}_{m}=\frac{I\times \Delta t}{m\times \Delta V}$$

Where $I$, $\,m$, $\Delta V$ and $\Delta t$ represent the charge/discharge current, the mass of the active materials in the single electrode (for the three-electrode system) and mass in both electrodes (for the two-electrode system), the potential window and the discharge time in the corresponding potential window, respectively.

In a two-electrode configuration, the symmetric EDLCs cell was fabricated by assembling two individual electrodes with the same mass loading of active materials. The potential that was applied to the EDLCs cell ranged from 0 to 1.6 V in 1 M Na_2_SO_4_ aqueous solution as the electrolyte.

According to the CV curves and the specific capacitance of EDLCs cell, *E* (energy density) and *P* (power density) of the symmetric EDLCs cell were calculated according to the equation:

$${C}_{sp}=4\times {C}_{m}$$

$$E=\frac{0.5\times {C}_{m}\times \Delta {V}^{2}}{3.6}$$

$$P=\frac{E}{\Delta t}\times 3600$$

Where ${C}_{sp}$ represents the specific capacitance of single electrode in the two-electrode cell, ${C}_{m}$ is calculated as previously mentioned. $\Delta V$ denotes the corresponding potential window except for IR drop in the discharge process and $\Delta t$ is the discharge time in the potential window.

## Supplementary information

Supplementary Information.
