# Supplementary material for: Ultrafast microwave synthesis of rambutan-like CMK-3/carbon nanotubes nanocomposites for high-performance supercapacitor electrode materials
Source: Sci Rep. 2020 Apr 10;10:6227. doi: 10.1038/s41598-020-63204-3 (PMC7148346; doi:10.1038/s41598-020-63204-3)
Supplement: Supplementary file 1 — Supplementary Information. [file 41598_2020_63204_MOESM1_ESM.pdf]

Supplementary Information

Ultrafast microwave synthesis of rambutan-like CMK-3/carbon nanotubes nanocomposites for high-performance supercapacitor electrode materials

Ke Yan, Xin Sun, Shu Ying, Wen Cheng, Yu Deng, Zhong Ma, Yu Zhao, Xinran Wang, Lijia Pan*, Yi Shi*

Jiangsu Provincial Key Laboratory of Photonic and Electronic Materials, Collaborative Innovation Center of Advanced Microstructures, School of Electronic Science and Engineering, Nanjing University, Nanjing 210093, China.

***** Corresponding author E-mail: ljpan@nju.edu.cn; yshi@nju.edu.cn.

Experimental Section

Synthesis of CMK-3

The hard-template synthesis of ordered mesoporous carbon CMK-3 follows the previously described method involving four main steps: 1. Embedding organic precursors into the molecular sieve templates SBA-15; 2. Polymerization; 3. Pyrolysis and carbonization; 4. Removal of SBA-15. In a typical process, 0.14g of sulfuric acid (98 wt.%) was dissolved in 5 g of deionized water, then 1.25 g of sucrose (95 wt.%) were added. SBA-15 (1 g, XF Nano) was impregnated with the solution and stirred for 30 minutes at room temperature. The mixture was heated in an oven at 100°C for 6 hrs, and subsequently, the temperature was raised to 160°C for 6 hrs. The resulted nanocomposites with dark brown color were impregnated once again with a mixture of aqueous solution of 0.82 g sulphuric acid, 0.09 g sucrose and 5 g of deionized water. After thorough mixing, the nanocomposites were re-treated at 100°C and 160°C for 6 hrs respectively to complete the whole polymerization step. In the pyrolysis and carbonization process, the nanocomposites were heated from room temperature to 900°C with a heating rate of 1°C·min^{-1} in an N_2 flow of 200 ml·min^{-1}. Then the temperature was kept for 3 hrs at 900°C and then naturally cooled down to room temperature. The removal of SBA-15 was conducted using hydrofluoric solutions (5 wt.% HF) to recover CMK-3 at room temperature for 24 hrs. After filtering, washing and drying, the CMK-3 powder was obtained.

Synthesis of SBA-15

Well-ordered hexagonal mesoporous silica structures SBA-15 with uniform pore diameters were synthesized as reported previously. Amphiphilic triblock copolymers (poly (ethylene glycol)-block-poly (propylene glycol)-block-poly (ethylene glycol) ($EO_{20}PO_{70}EO_{20}$, MW= 5800 $g \cdot mol^{-1}$, Aldrich)) were used to help the organization of polymerizing silica species in acidic media. Typically, 4 g of the amphiphilic triblock copolymer was dispersed in 30 g of water at 25°C and 120 g of 2 M HCl solution while stirring. After 5 hrs of stirring, 9.5 g of tetraethyl orthosilicate (TEOS) was added to the homogeneous solution and keep stirring vigorously for another 6 hrs. The resulting gel was aged at 40°C for 18 hrs and then heated to the crystallizing temperature between 100°C and 150°C for 24 hrs. Finally, the resulted species were annealed at 540°C in air. As-synthesized SBA-15 has the pore diameters ranging from 9.0 nm to 11.2 nm.

Pore diameter control of CMK-3

The control of CMK-3 channel size was performed by synthesizing CMK-3 with tunable pore sizes. The precursor aqueous solution of sucrose and various amount of boric acid was added. Boric acid act as pore expansion agent which adhere on the SBA-15 pore wall to adjust the pore size and carbon cylinder diameter simultaneously. The mesoporous carbon replicas of the SBA-15 silica templates were designated as CMK-3 x (x denotes a, b, c…), indicating the different molar ratio of boric acid to sucrose. A typical procedure of CMK-3 b was performed as follows: 0.113 g of boric acid, 0.625 g of sucrose and 0.07 g sulfuric acid were

dissolved in 2.5 g of deionized water. After that, 0.5 g of SBA-15 molecular sieve templates were added in the homogeneous solution and transferred to a hydrothermal reactor to be heated at 100°C for 6 hrs, and then at 160°C for another 6 hrs. The resulted mixture was added again with an aqueous solution consisting of 0.15 g of boric acid, 0.41 g of sucrose, 0.045 g of sulfuric acid and 2.5 g of deionized water. After retreated at 100°C and 160°C for 6 hrs respectively, the resulted mixture was carbonized at 900°C for 3 hrs under N_2 flow. The CMK-3 was recovered by removing the mesoporous framework in a 5 wt.% hydrofluoric solution at room temperature for 24 hrs. The obtained carbon, without a template, was filtered and washed several times with deionized water and ethanol (50:50% v/v) and overnight dried at 60°C.

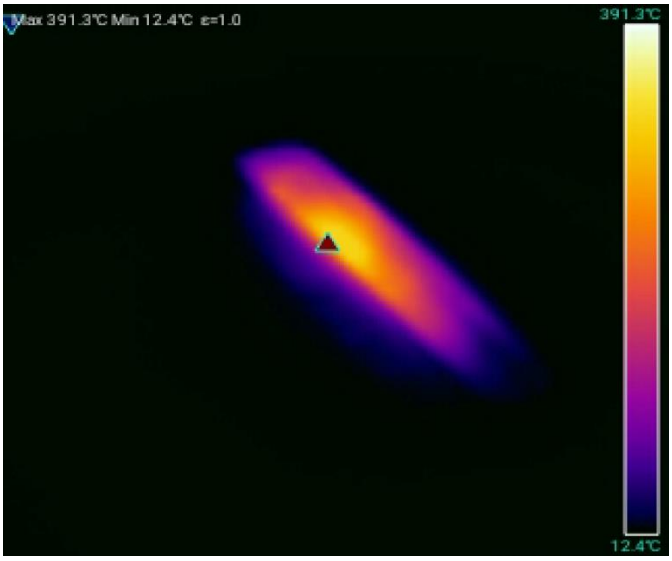

Figure S1 Thermal imaging of the sample after about 10 seconds' microwave irradiation, the temperature measured ranging from 12.4°C to 391.3°C (room temperature was about 12°C).

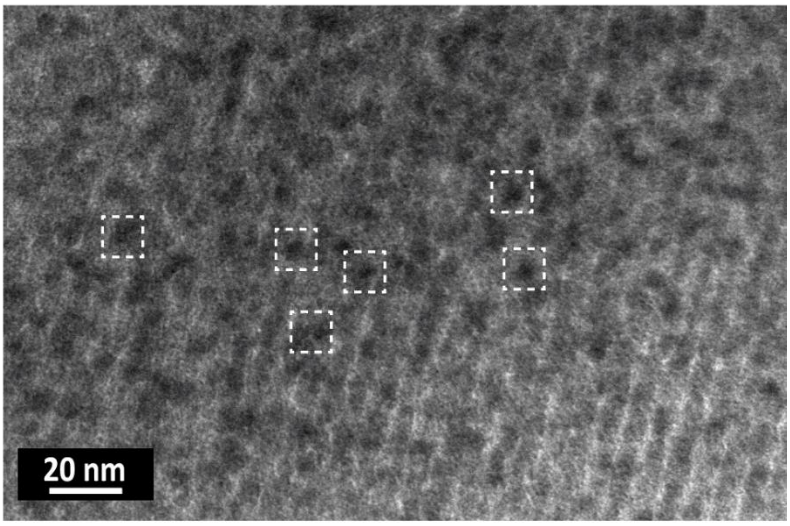

Figure S2 TEM image of CMK–3 after the microwave process, in which Fe_2O_3 nanoparticles (marked by white case) are embedded and well dispersed in the nanochannels of CMK–3. The formation of Fe_2O_3 was ascribed to the oxidization in air of Fe nanoparticles under high heating temperature.

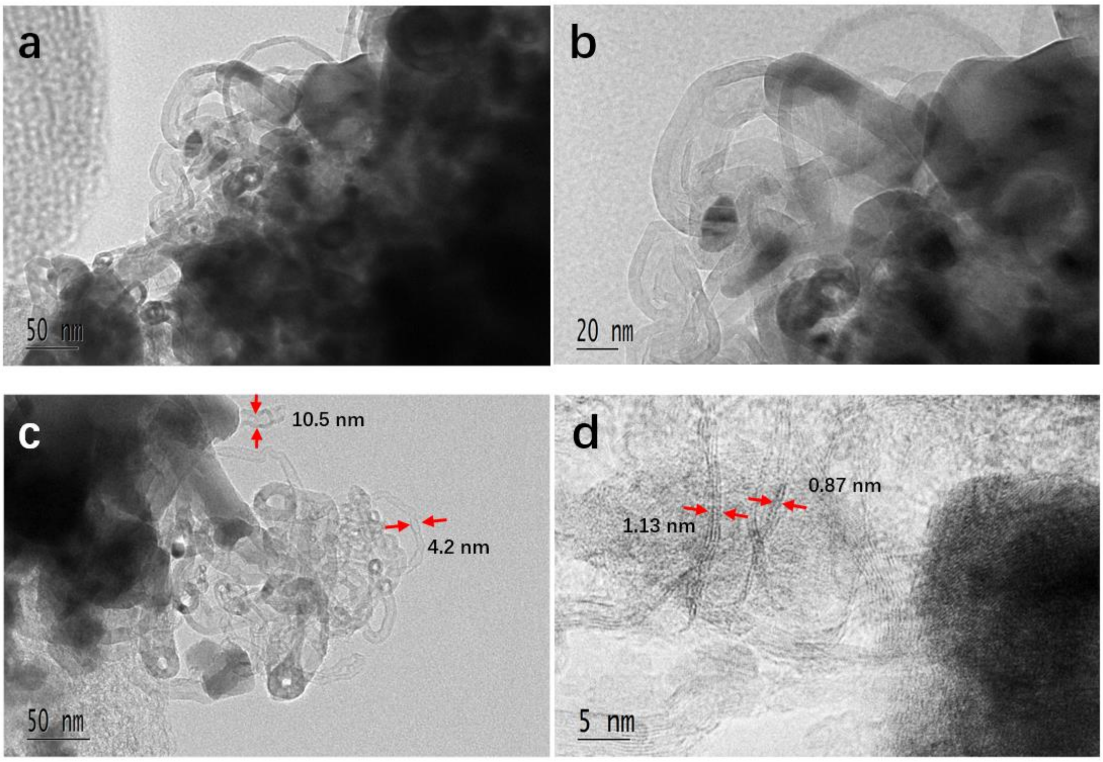

Figure S3 TEM image of CMK–3/CNTs nanocomposites after microwave induced CNTs synthesis: (a) and (b) CNTs grow into bundles and wrapped on the surface of CMK-3; (c) Highly dense of CNTs with diameter ranging from ~ 4 nm to ~ 10 nm were formed; (d) Single wall carbon nanotubes with diameter of ~1 nm were formed by the catalyst confined in the CMK-3 channels.

Table S1. Catalyst concentrations and ID/IG values of as-synthesized CMK-3/CNTs nanocomposites.

Sample	Catalyst concentration (%)	I_D/I_G
CMK-3/CNTs-60%	5.49	0.72
CMK-3/CNTs-70%	6.16	0.61
CMK-3/CNTs-80%	7.29	0.50
CMK-3/CNTs-90%	9.95	0.51
CMK-3/CNTs-100%	18.8	0.64

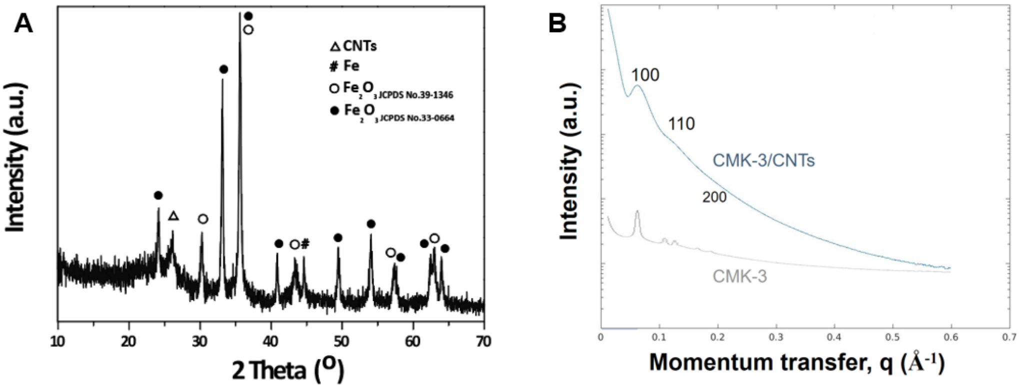

Figure S4 A. The XRD patterns of the CMK-3/CNTs nanocomposites before acid treatment process which shows the co-existence of CNTs, Fe_2O_3 and Fe. The diffraction peaks could be assigned to graphite CNTs, Fe (JCPDS06-0696) and Fe_2O_3 (JCPDS No.33-0664 and No.39-1346) nanoparticles embedded in the CMK-3 nanochannels; B. SAXS patterns of CMK-3 and CMK-3/CNTs nanocomposites. The peaks of 100, 110, 200 illustrates the hexagonal array (p6mm) of cylindrical pores. The d-spacing values are calculated by the formula $d = 2\pi /q$ and unit cell parameters are calculated by the formula $a = 2 \cdot 3^{-2} \cdot d_{100}$. The unit cell parameters of CMK-3 and composites are calculated to be ~11.3 nm.

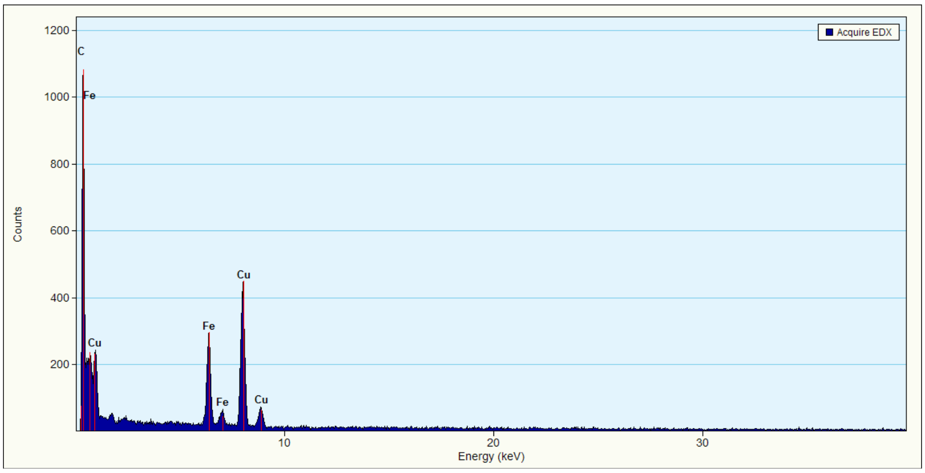

Figure S5 Energy Dispersive X-Ray spectroscopy (EDX) pattern of CMK-3/CNTs nanocomposites.

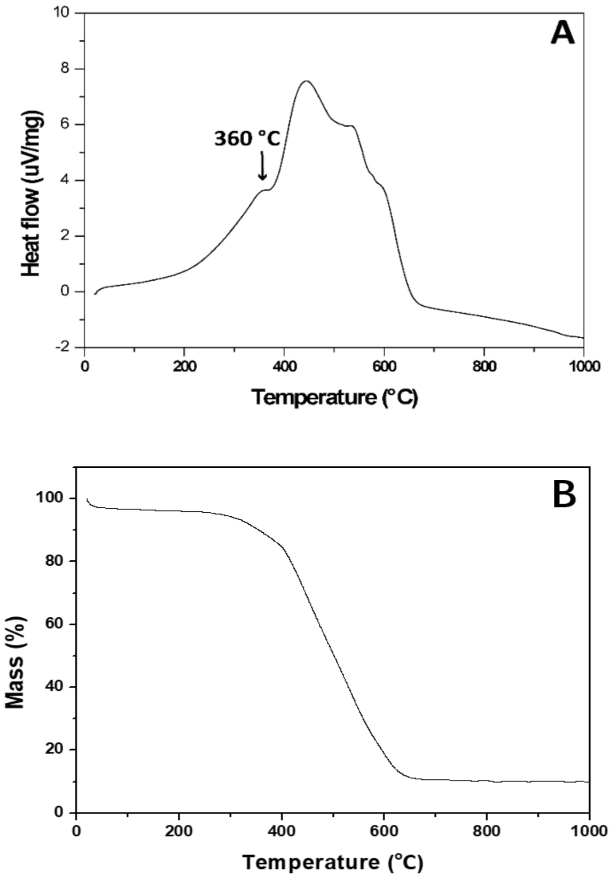

Figure S6 A. Differential Scanning calorimeter (DSC) curve of the precursor compounds (heating rate: 10°C/min); B. Thermogravimetric curve of the CMK-3/CNTs nanocomposites (heating rate: 10°C/min).

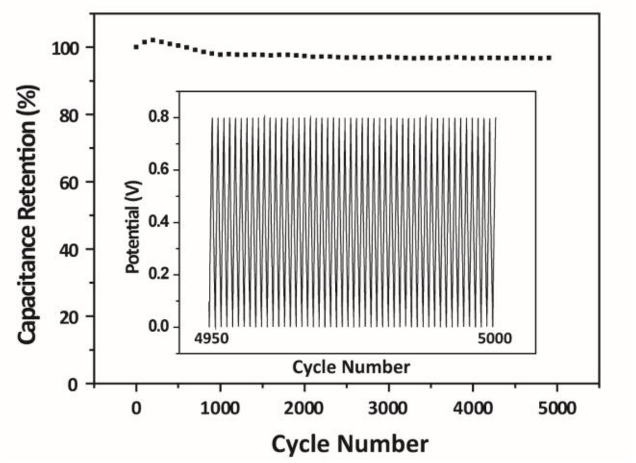

Figure S7 Cycling performance of CMK-3/CNTs nanocomposites at current density of 10 A·g-1; Inset, the GCD curves between 4950th and 5000th cycle.

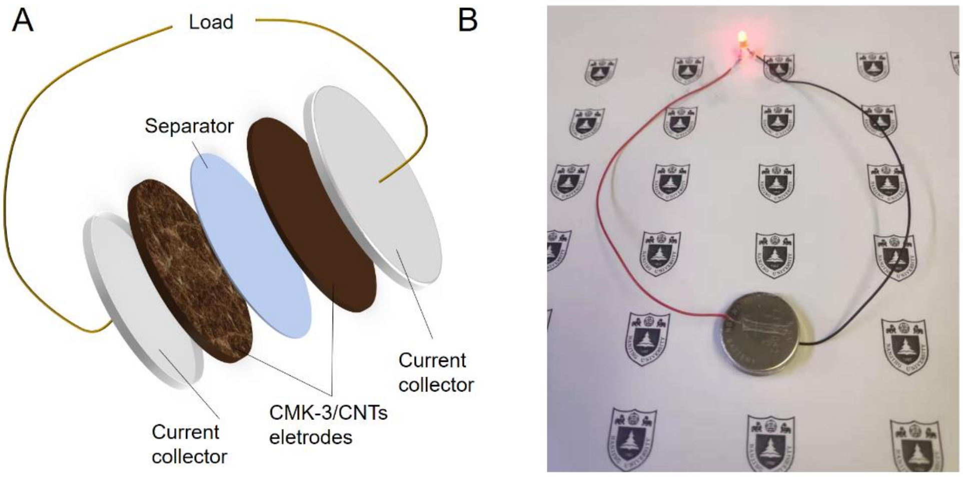

Figure S8 A. Schematic illustration and photograph of the supercapacitor prototype; B. Fully packaged coin-type supercapacitors of 2 symmetric supercapacitors connected in series can power a red light-emitting diodes (LED) light.

Table S2. Comparison of electrode materials recently applied for supercapacitor applications and as-made asymmetric/symmetric supercapacitors.

Materials	specific capacitance	rate capability	energy density	cycling capacitance retention (%)	ref.
CMK-3/CNTs	315.6 $F \cdot g^{-1}$ at 1 $A \cdot g^{-1}$	214.6 $F \cdot g^{-1}$ at 50 $A \cdot g^{-1}$	21.7 $Wh \cdot kg^{-1}$ at 411.7 $W \cdot kg^{-1}$	99.32% after 10,000 cycles	This work
Graphene Hydrogels	220 $F \cdot g^{-1}$ at 1 $A \cdot g^{-1}$	165 $F \cdot g^{-1}$ at 50 $A \cdot g^{-1}$	5.7 $Wh \cdot kg^{-1}$ at 30 $kW \cdot kg^{-1}$	92% after 2,000 cycles	1
Hollow carbon spheres/carbon nanotubes	201.5 $F \cdot g^{-1}$ at 0.5 $A \cdot g^{-1}$	139 $F \cdot g^{-1}$ at 20 $A \cdot g^{-1}$	11.3 $Wh \cdot kg^{-1}$ at 127 $W \cdot kg^{-1}$	90% after 5,000 cycles	2
Flower-like porous carbon	294 $F \cdot g^{-1}$ at 2 $mV \cdot s^{-1}$	209 $F \cdot g^{-1}$ at 500 $mV \cdot s^{-1}$	15.9 $Wh \cdot kg^{-1}$ at 317.5 $W \cdot kg^{-1}$	98% after 5,000 cycles	3
N&S co-doped C nanosheets	298 $F \cdot g^{-1}$ at 0.5 $A \cdot g^{-1}$	233 $F \cdot g^{-1}$ at 50 $A \cdot g^{-1}$	21.0 $Wh \cdot kg^{-1}$ at 180 $W \cdot kg^{-1}$	98% after 10,000 cycles	4
(PS-b-PEO) carbon spheres	170 $F \cdot g^{-1}$ at 1 $A \cdot g^{-1}$	90 $F \cdot g^{-1}$ at 10 $A \cdot g^{-1}$	12.5 $Wh \cdot kg^{-1}$	NA	5
Flexbile 3D graphene hydrogel	186 $F \cdot g^{-1}$ at 1 $A \cdot g^{-1}$	130.2 $F \cdot g^{-1}$ at 20 $A \cdot g^{-1}$	6.1 $Wh \cdot kg^{-1}$ at ~1.1 $kW \cdot kg^{-1}$	91.6% after 10,000 cycles	6
NiO/graphene foam	116 $F \cdot g^{-1}$ at 1 $A \cdot g^{-1}$	61 $F \cdot g^{-1}$ at 60 $A \cdot g^{-1}$	17 $Wh \cdot kg^{-1}$ at 42 $kW \cdot kg^{-1}$	94% after 2,000 cycles	7
α-Fe_2O_3 nanotubes/rGO	181 $F \cdot g^{-1}$ at 3 $A \cdot g^{-1}$	69 $F \cdot g^{-1}$ at 10 $A \cdot g^{-1}$	17 $Wh \cdot kg^{-1}$ at 42 $kW \cdot kg^{-1}$	92% after 2,000 cycles	8
Layer-by-layer MnO_2/rGO	286 $F \cdot g^{-1}$ at 3 $A \cdot g^{-1}$	169 $F \cdot g^{-1}$ at 10 $A \cdot g^{-1}$	42 $Wh \cdot kg^{-1}$ at 7.6 $kW \cdot kg^{-1}$	79% after 10,000 cycles	9
δ-MnO_2/ graphene hybrid	267 $F \cdot g^{-1}$ at 0.2 $A \cdot g^{-1}$	208 $F \cdot g^{-1}$ at 10 $A \cdot g^{-1}$	18.64 $Wh \cdot kg^{-1}$ at 12.6 $kW \cdot kg^{-1}$	92% after 7,000 cycles	10
2D Co_3O_4 3D nanoflakes	1500 $F \cdot g^{-1}$ at 1 $A \cdot g^{-1}$	828 $F \cdot g^{-1}$ at 10 $A \cdot g^{-1}$	15.4 $Wh \cdot kg^{-1}$ at 0.8 $kW \cdot kg^{-1}$	99.3% after 2,000 cycles	11
MnO_2/Ti_3C_2Tx hybrid	212.1 $F \cdot g^{-1}$ at 1 $A \cdot g^{-1}$	176 $F \cdot g^{-1}$ at 40 $A \cdot g^{-1}$	12.25 $Wh \cdot kg^{-1}$ at 20 $kW \cdot kg^{-1}$	88% after 2,000 cycles	12
TiO_2/Ti_3C_2 nanocomposite	143 $F \cdot g^{-1}$ at 5 $mV \cdot s^{-1}$	117 $F \cdot g^{-1}$ at 200 $mV \cdot s^{-1}$	NA	92% after 6,000 cycles	13

References:

1. Zhang, L. & Shi, G. Preparation of Highly Conductive Graphene Hydrogels for Fabricating Supercapacitors with High Rate Capability. J. Phys. Chem. C 115, 17206-17212 (2011)
2. Wang, Q. *et al.* Template synthesis of hollow carbon spheres anchored on carbon nanotubes for high rate performance supercapacitors. *Carbon* **52**, 209-218 (2013)
3. Wang, Q. *et al.* Three-dimensional flower-like and hierarchical porous carbon materials as high-rate performance electrodes for supercapacitors. *Carbon* **67**, 119-127 (2014).
4. Li, Y., Wang, G., Wei, T., Fan, Z. & Yan, P. Nitrogen and sulfur co-doped porous carbon nanosheets derived from willow catkin for supercapacitors. *Nano Energy* **19**, 165-175 (2016).
5. Tang, J. *et al.* Activated Porous Carbon Spheres with Customized Mesopores through Assembly of Diblock Copolymers for Electrochemical Capacitor. *ACS Appl Mater Interfaces* **9**, 18986-18993 (2017).
6. Xu, Y. *et al.* Flexible solid-state supercapacitors based on three-dimensional graphene hydrogel films. *ACS Nano* **7**, 4042-4049 (2013).
7. Wang, H., Yi, H., Chen, X. & Wang, X. Asymmetric supercapacitors based on nano-architectured nickel oxide/graphene foam and hierarchical porous nitrogen-doped carbon nanotubes with ultrahigh-rate performance. *J. Mater. Chem. A* **2**, 3223-3230 (2014).
8. Lee, K. K. *et al.* alpha-Fe2O3 nanotubes-reduced graphene oxide composites as synergistic electrochemical capacitor materials. *Nanoscale* **4**, 2958-2961 (2012).
9. Jana, M. *et al.* A successive ionic layer adsorption and reaction (SILAR) method to fabricate a layer-by-layer (LbL) MnO2 -reduced graphene oxide assembly for supercapacitor application. *Journal of Power Sources* **340**, 380-392 (2017).
10. Peng, L. *et al.* Ultrathin two-dimensional MnO2/graphene hybrid nanostructures for high-performance, flexible planar supercapacitors. Nano Letter **13**, 2151−2157 (2013).
11. Jiang, Y. *et al.* Two-dimensional Co3O4 thin sheets assembled by 3D interconnected nanoflake array framework structures with enhanced supercapacitor performance derived from coordination complexes. Chem. Eng. J. **292**, 1−12 (2016).
12. Rakhi, R. B., Ahmed, B., Anjum, D., Alshareef, H. N. Direct chemical synthesis of MnO2 nanowhiskers on transition metal carbide surfaces for supercapacitor applications. ACS Appl. Mater. Interfaces **8**, 18806−18814 (2016).
13. Zhu, J., Tang, Y., Yang, C., Wang, F. & Cao, M. Composites of TiO2 nanoparticles deposited on Ti3C2 MXene nanosheets with enhanced electrochemical performance. J. Electrochem. Soc. **163**, A785−A791 (2016).
